# A comparison of the antianhedonic effects of repeated ketamine infusions in melancholic and non-melancholic depression

**DOI:** 10.3389/fpsyt.2022.1033019

**Published:** 2022-12-22

**Authors:** Wei Zheng, Xin-Hu Yang, Li-Mei Gu, Jian-Qiang Tan, Yan-Ling Zhou, Cheng-Yu Wang, Yu-Ping Ning

**Affiliations:** ^1^The Affiliated Brain Hospital of Guangzhou Medical University, Guangzhou, China; ^2^The First School of Clinical Medicine, Southern Medical University, Guangzhou, Guangdong, China

**Keywords:** clinical trial, ketamine, depression, melancholia, response

## Abstract

**Objectives:**

Melancholic depression may respond differently to certain treatments. The aim of this study was to compare the antianhedonic effects of six intravenous injections of 0.5 mg/kg ketamine in patients with melancholic and non-melancholic depression, which remain largely unknown.

**Methods:**

Individuals experiencing melancholic (*n* = 30) and non-melancholic (*n* = 105) depression were recruited and assessed for anhedonic symptoms using the Montgomery–Åsberg Depression Rating Scale (MADRS). The presence of melancholic depression was measured with the depression scale items at baseline based on DSM-5 criteria.

**Results:**

A total of 30 (22.2%) patients with depression fulfilled the DSM-5 criteria for melancholic depression. Patients with melancholic depression had a non-significant lower antianhedonic response (43.3 vs. 50.5%, *t* = 0.5, *p* > 0.05) and remission (20.0 vs. 21.0%, *t* = 0.01, *p* > 0.05) to repeated-dose ketamine infusions than those with non-melancholic depression. The melancholic group had significantly lower MADRS anhedonia subscale scores than the non-melancholic group at day 26 (*p* < 0.05).

**Conclusion:**

After six ketamine infusions, the improvement of anhedonic symptoms was found in both patients with melancholic and non-melancholic depression, and the efficacy was similar in both groups.

## Introduction

Melancholic features were classified by the Diagnostic and Statistical Manual of Mental Disorders 5th edition (DSM-5) as a particular subtype of major depressive disorder (MDD), which may coexist with other patterns of depressive symptoms (1). This concept of melancholic depressive symptoms primarily originated from the historic concept of “endogenous depression” (2). Melancholia can occur in either MDD or major depressive episodes (MDEs) of bipolar depression (BD) (3). Melancholic depression may be associated with a relatively severe clinical manifestation of mood disorder (3).

Patients with melancholic depressive are differentiated from patients with non-melancholic depressive with regard to clinical characteristics, neurocognitive dysfunctions, treatment response patterns, and neuroimaging findings. For example, a recent meta-analysis found that acute episodes of MDD with melancholic features had greater neurocognitive deficits than episodes with non-melancholic features (4). A prospective study reported that subjects with melancholic features have higher excess all-cause mortality than those without melancholic features (5). However, findings on the treatment responses to psychotropic drug treatments between patients with melancholic and non-melancholic features were inconsistent. For example, when compared to those with non-melancholic depression, patients with melancholic depression had similar responses to antidepressants (3, 6) and quetiapine (7), had stronger responses to lithium (8) and electroconvulsive therapy (ECT) (9), and had weaker responses to psychotherapy (10).

Apart from the rapid and robust antisuicidal and antidepressant effects (11–15), accumulating evidence has shown that both single and repeated ketamine injections at a subanaesthetic dose (0.5 mg/kg over 40 min) have rapid and robust antianhedonic effects in individuals suffering from MDD and BD (16–19). Notably, ketamine's antianhedonic effects were independent of other depressive symptoms (16). A recent study found that a single dose of ketamine appears to be effective in treating both melancholic/typical and atypical depressive symptoms (1). However, the differences in the antianhedonic effects of repeated ketamine infusions in patients with melancholic and non-melancholic depression have remained unknown.

In this exploratory study, we divided the participants into melancholic and non-melancholic subtypes and sought to comparatively investigate the antianhedonic effects of multiple intravenous injections of 0.5 mg/kg ketamine in individuals with melancholic and non-melancholic depression. Based on the findings of a recent study (1), we hypothesized that multiple intravenous injections of ketamine effectively treated anhedonic symptoms in both melancholic and non-melancholic depression.

## Methods

In this *post hoc* analysis, data were drawn from an ongoing real-world open-label study investigating the efficacy and safety of adjunctive multiple ketamine infusions for the treatment of patients with depression with TRD and/or suicidal ideation, which was initiated in November 2016 and registered in the Chinese Clinical Trail Registry (Clinical Trials Identifier: ChicCTR-OOC-17012239). All patients provided written informed consent, and approval was obtained from the Affiliated Brain Hospital of Guangzhou Medical University respective Institutional Review Board (IRB) (Ethical Application Ref: 2016030).

### Patients

Patients with depression were recruited from the Affiliated Brain Hospital of Guangzhou Medical University. The inclusion criteria for this real-world open-label study were as follows: (1) a diagnosis of MDD or BD without hallucinations or delusions according to the DSM-5 criteria; (2) experiencing a MED with a baseline score ≥17 on the Hamilton Depression Rating Scale-17 (HAMD-17); (3) aged 18–65 years; and (4) suffering from suicidal ideation with a Beck Scale for Suicide Ideation-part I (SSI-part I) scores of 2 or higher and/or TRD, defined as having failed attempts to achieve a response to two trials of antidepressants. The exclusion criteria were as follows: (1) patients fulfilling the DSM-5 criteria for other serious mental disorders, such as schizophrenia or alcohol/substance use disorder; (2) patients with a positive urine toxicology screen; (3) patients with any serious or unstable somatic diseases, such as cancer or infectious disease; and (4) patients who were pregnant or breast feeding.

### Repeated-dose ketamine infusions

The procedures for subanaesthetic intravenous ketamine have been detailed previously (11). In brief, as recommended previously (12), all participants received a course of six intravenous infusions of ketamine hydrochloride (0.5 mg/kg over 40 min) administered thrice weekly over the course of 2 weeks following overnight fasting. A psychiatrist recorded vital signs, including pulse frequency, blood pressure and heart rate, every 10 min throughout the infusion and monitoring period. All subjects remained on stable type and dosage of concomitant psychotropic medication during the infusion treatment.

### Clinical interview and assessments

A detailed demographic questionnaire was conducted for patients with melancholic and non-melancholic depression, recording general information and socio-demographic characteristics, such as age, gender, and marital status. Clinical ratings of the severity of anhedonic symptoms measured in a sample of individuals with melancholic and non-melancholic depression at baseline, at 4 and 24 h after each infusion of the study agent, and at 2 weeks postinfusion (day 26) using the Montgomery–Åsberg Depression Rating Scale (MADRS). Following the methodology of previous studies (20–22), the anhedonia items of the MADRS, including assessments of apparent sadness, concentration difficulties, lassitude, reported sadness, and inability to feel, were utilized to assess the severity of anhedonic symptoms (23, 24). The coprimary endpoints were the comparison of antianhedonic response and remission (≥50 and ≥75% reduction of the MADRS anhedonia subscale scores at day 13, respectively) (25, 26) between individuals with melancholic and non-melancholic depression. The secondary endpoint was the comparison of the severity of anhedonic symptoms between individuals with melancholic and non-melancholic depression. The intraclass correlation coefficient (ICC) for the MADRS anhedonia subscale scores was >0.9, suggesting excellent interrater reliability.

### Definition of melancholic depression

As recommended previously (7), baseline scores on the HAMD-17 and MADRS were used to split the population into two subgroups (patients with melancholic and non-melancholic depression). The presence of melancholic depression was defined based on DSM-5 criteria (7), which require anhedonia in nearly all activities (MADRS item 8 ≥ 4) and/or non-reactive mood (MADRS items 1 or 2 = 6), and at least three of the following: significant psychomotor retardation or agitation (HAMD-17 items 8 or 9 ≥ 2), marked appetite/weight loss (HAMD-17 items 12 or 16 = 2), terminal insomnia (HAMD-17 item 6 ≥ 1), and unwarranted or disproportionate guilt (HAMD-17 item 2 ≥ 2).

### Statistical analysis

In this study, we used SPSS version 24.0 (SPSS Inc., Chicago, United States) for all statistical analyses. Intent-to-treat analysis was conducted in this study. The demographic and clinical variables of individuals with melancholic and non-melancholic depression were compared with Student's *t*-test for continuous variables (including age, body mass index, education, depressive symptoms, anxiety symptoms, and suicidal ideation) and the Chi-square test for categorical variables (including gender, marital status, and rates of antianhedonic response, and remission). The rates of antianhedonic response and remission between individuals with melancholic and non-melancholic depression were analyzed by the Chi-square test. The comparisons of the rates of antianhedonic response and remission between the two groups were performed using odds ratios derived from logistic regression analyses after adjusting for the sociodemographic confounding variables. A linear mixed-effects model was utilized to determine the difference in anhedonic symptoms over time between groups. The covariates in the linear mixed-effects model analysis included baseline demographic and clinical variables that differed between the two groups. We utilized Bonferroni correction to adjust for multiple comparisons and set the significance level α at 0.05.

## Results

### Demographics of the non-melancholic and melancholic groups

Among 135 patients with depression receiving repeated ketamine infusions, 30 (22.2%) fulfilled the DSM-5 criteria for melancholic depression, and 105 (77.8%) did not. The demographic and clinical characteristics of patients with melancholic depression vs. non-melancholic depression are summarized in Table 1. As expected, the melancholic subgroup had higher baseline HAMD-17 scores (*t* = 10.5, *p* < 0.001), baseline MADRS scores (*t* = 7.8, *p* < 0.001), and baseline MADRS anhedonia subscale scores (*t* = 17.4, *p* < 0.001) (Table 1). The subgroups did not differ with regard to age, sex, education level, or age of onset (all *p* > 0.05).

**Table 1:** Comparison of demographic and clinical characteristics between patients with melancholic depression and non-melancholic depression.

**Variables**	**Melancholic (*****n*** = **30)**		**Non-melancholic** **(*****n*** = **105)**		**Statistics**		
	***N***	**%**	***N***	**%**	χ^2^	***df***	***P***
Male	12	40.0	56	53.3	1.7	1	0.19
Married	17	56.7	61	58.1	0.02	1	0.89
Employed	13	43.3	39	37.1	0.4	1	0.54
Living alone	2	6.7	9	8.6	0.1	1	0.74
No history of psychiatric hospitalization	19	63.3	74	70.5	0.6	1	0.46
Having a family history of psychiatric disorders	14	46.7	38	36.2	1.1	1	0.29
Antianhedonic responders	13	43.3	53	50.5	0.5	1	0.49
Antianhedonic remitters	6	20.0	22	21.0	0.01	1	0.91
	**Mean**	**SD**	**Mean**	**SD**	**T/Z**	**df**	***P***
Age (years)	34.7	10.5	34.8	12.1	0.05	133	0.96
Education (years)	12.1	3.9	12.2	3.1	−0.2	133	0.87
BMI (kg/m^2^)	22.4	3.4	22.7	3.5	−0.3	133	0.73
Age of onset (years)	26.6	11.4	26.1	11.6	0.2	133	0.83
Duration of illness (months)	94.4	85.5	104.2	93.8	−0.5	133	0.61
Baseline HAMD-17 scores	30.2	4.7	21.9	3.5	10.5	133	<**0.001**
Baseline MADRS scores	41.0	6.7	30.4	6.5	7.8	133	<**0.001**
Baseline MADRS anhedonia subscale scores	24.2	2.6	19.3	4.6	5.5	133	<**0.001**
HAMD-17 scores at post-treatment	13.9	8.1	11.3	7.0	1.7	133	0.09
MADRS scores at post-treatment	20.4	12.2	15.4	10.9	2.2	133	**0.03**

Bolded values are p < 0.05. BMI, body mass index; HAMD, Hamilton Depression Rating Scale; MADRS, Montgomery–Åsberg Depression Rating Scale.

### Antianhedonic response and remission between the non-melancholic and melancholic groups

As shown in Table 1, patients with non-melancholic depression had significantly lower MADRS scores at post-treatment (15.4 ± 10.9 vs. 20.4 ± 12.2, *p* < 0.05) than those with melancholic depression, but significance disappeared after controlling for baseline MADRS scores (*p* > 0.05). Patients with non-melancholic depression had non-significantly lower HAMD scores at post-treatment (11.3 ± 7.0 vs. 13.9 ± 8.1, *p* > 0.05) than those with melancholic depression. Patients with melancholic depression achieved a non-significant lower antianhedonic response to repeated-dose ketamine infusions than those with non-melancholic depression [43.3% (13/30) vs. 50.5% (53/105), *t* = 0.5, *p* > 0.05]. Similarly, patients with melancholic depression met a non-significant lower antianhedonic remission criteria than those with non-melancholic depression [20.0% (6/30) vs. 21.0% (22/105), *t* = 0.01, *p* > 0.05]. No significant differences between the two groups were observed regarding antianhedonic response and remission rates after controlling for confounds (all *p* > 0.05).

### Anhedonic symptoms between the non-melancholic and melancholic groups

**Figure 1:**
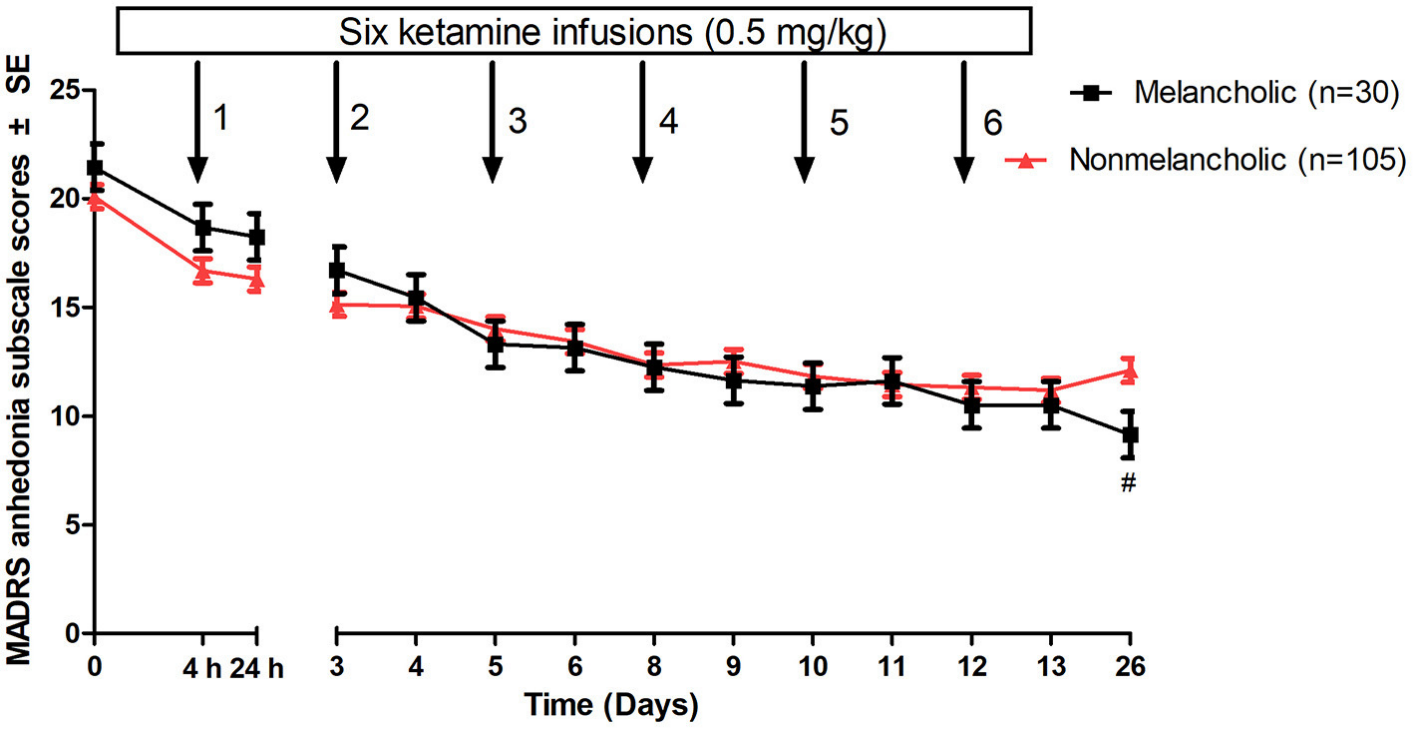
Change in ahedonic symptoms in patients with melancholic and non-melancholic depression following multiple ketamine infusions. Values covaried for baseline MADRS anhedonia subscale scores. ^#^A significant difference was found at a given time point between patients with melancholic and non-melancholic depression (*p* < 0.05). MADRS, Montgomery-Åsberg Depression Rating Scale; SE, standard error.

The linear mixed model with MADRS anhedonia subscale scores showed significant main effects for group-by-time interaction (*F* = 3.0, *p* < 0.001) and time (*F* = 64.4, *p* < 0.001) but not for group (*F* = 0.6, *p* = 0.46). Compared with baseline, significant improvements in anhedonic symptoms were found from day 1 to 26 and from day 3 to 26 in the non-melancholic and melancholic groups, respectively (all *p* < 0.05). As shown in Figure 1, the melancholic group had significantly lower MADRS anhedonia subscale scores than the non-melancholic group at day 26 (*p* < 0.05).

## Discussion

To the best of our knowledge, this is the first study to examine the differences in antianhedonic response and remission to six intravenous injections of 0.5 mg/kg ketamine over 40 min in individuals with non-melancholic and melancholic depression. The following major findings were obtained: (1) 22.2% (30/135) of subjects reported melancholic depression; (2) similar antianhedonic response and remission rates were found in individuals with or without melancholic depression after six injections of ketamine; and (3) the reduction of anhedonic symptoms in patients with melancholic depression was greater at day 26 than in patients with non-melancholic depression.

Based on the DSM-5 criteria, 22% of participants suffer from melancholic depression, which is relatively lower than the figure (31.7%) reported in a previous study (7). Another study (27) found that 13 of 33 (39.3%) participants were classified as having melancholic depression according to the CORE measure (28). The differences in the presence of melancholic depression between our findings and Spanemberg et al.'s study (27) are mainly attributed to differential diagnosis criteria for melancholic depression. Furthermore, Joyce et al. found that the CORE criteria for melancholia, but not the Diagnostic and Statistical Manual of Mental Disorders 4th edition (DSM-IV), had greater neuroendocrine dysfunction (29).

In this *post hoc* secondary analysis, significant rapid improvements in anhedonic symptoms in both patients with and without melancholic depression were observed in response to six ketamine infusions in this group of individuals suffering from either MDD or BD. Furthermore, the antianhedonic response and remission to repeated intravenous administration of subanaesthetic doses of ketamine were similar in patients with and without melancholic depression. The fact that this difference did not achieve statistical significance may be due to the relatively small number of melancholic patients in the sample. The potential for a superior result with melancholic patients deserves further study with a larger sample. Similarly, a single ketamine infusion effectively reduced depressive symptoms in patients with melancholic/typical and atypical depression, with similar efficacy in both groups (1). However, the differences in antianhedonic effects of a single ketamine infusion between the two groups should be investigated in future studies.

The present study has several strengths and limitations. The largest strength of this study is that it is the first to compare the antianhedonic effects of ketamine between patients with melancholic depression and those with non-melancholic depression. The limitations of this study are as follows: (1) open-label design; (2) the sample size for the melancholic group (*n* = 30) was relatively small; (3) the pooling of individuals with MDD and BD increasing sample heterogeneity; (4) the anhedonia items of the MADRS were used to assess anhedonic symptoms rather than a specific scale for anhedonia, such as the Snaith–Hamilton Pleasure Scale (SHAPS) (30–32); and (5) the secondary/*post hoc* analysis of melancholic depression based on scale items.

## Conclusion

After six ketamine infusions, an improvement in anhedonic symptoms was observed in patients with melancholic and non-melancholic depression, but with similar efficacy in both groups. These findings are still exploratory, and future studies with a randomized, active placebo-controlled design are warranted.

## Significant outcomes

- The prevalence of melancholic depression was 22.2%.
- Ketamine effectively relieved anhedonic symptoms in both patients with melancholic and non-melancholic depression.
- The antianhedonic effects of ketamine was similar in patients with melancholic than non-melancholic depression.

## Limitations

- This study was a *post hoc* secondary analysis.
- Participants were pooled across diagnoses (bipolar depression and major depressive disorder).
- This study was conducted based on an open-label design.

## Data availability statement

The original contributions presented in the study are included in the article/supplementary material, further inquiries can be directed to the corresponding authors.

## Ethics statement

The studies involving human participants were reviewed and approved by the Affiliated Brain Hospital of Guangzhou Medical University respective Institutional Review Board (IRB) (Ethical Application Ref: 2016030). The patients/participants provided their written informed consent to participate in this study.

## Author contributions

Y-PN: study design and critical revision of the manuscript. WZ, Y-LZ, and C-YW: data collection. WZ, X-HY, and L-MG: analysis and interpretation of data. WZ and J-QT: drafting of the manuscript. All authors: approval of the final version for publication.
